# FGFR Inhibitors in Oncology: Insight on the Management of Toxicities in Clinical Practice

**DOI:** 10.3390/cancers13122968

**Published:** 2021-06-13

**Authors:** Anuhya Kommalapati, Sri Harsha Tella, Mitesh Borad, Milind Javle, Amit Mahipal

**Affiliations:** 1 Department of Medical Oncology, Mayo Clinic, Rochester, MN 55905, USA; kommalapati.anuhya@mayo.edu (A.K.); tella.sri@mayo.edu (S.H.T.); 2 Department of Medical Oncology, Mayo Clinic, Phoenix, AZ 85259, USA; Borad.Mitesh@mayo.edu; 3 Department of Gastrointestinal Oncology, University of Texas MD Anderson Cancer Center, Houston, TX 77030, USA; mjavle@mdanderson.org

**Keywords:** FGFR, hyperphosphatemia, urothelial carcinoma, cholangiocarcinoma, pemigatinib, erdafitinib

## Abstract

**Simple Summary:**

FGFR inhibitors evolved as therapeutic options in cholangiocarcinoma and urothelial malignancies. Given the implications of FGFR pathway in various physiological functions, FGFR inhibitors are known to cause unique toxicities. In this review, we summarized the physiology of FGF/FGFR signaling and briefly discussed the possible mechanisms that could lead to FGFR inhibitor resistance and side effects. In addition, we proposed treatment guidelines for the management of FGFR-inhibitor-associated toxicities.

**Abstract:**

Fibroblast Growth Factor receptor (FGFR) pathway aberrations have been implicated in approximately 7% of the malignancies. As our knowledge of FGFR aberrations in cancer continues to evolve, FGFR inhibitors emerged as potential targeted therapeutic agents. The promising results of pemigatinib and infigratinib in advanced unresectable cholangiocarcinoma harboring *FGFR2* fusions or rearrangement, and erdafitinib in metastatic urothelial carcinoma with *FGFR2* and *FGFR3* genetic aberrations, lead to their accelerated approval by the United States (USA) FDA. Along with these agents, many phase II/III clinical trials are currently evaluating the use of derazantinib, infigratinib, and futibatinib either alone or in combination with immunotherapy. Despite the encouraging results seen with FGFR inhibitors, resistance mechanisms and side effect profile may limit their clinical utility. A better understanding of the unique FGFR-inhibitor-related toxicities would invariably help us in the prevention and effective management of FGFR-inhibitor-induced adverse events thereby enhancing their clinical benefit. Herein, we summarized the physiology of FGF/FGFR signaling and briefly discussed the possible mechanisms that could lead to FGFR inhibitor resistance and side effects. In addition, we proposed treatment guidelines for the management of FGFR-inhibitor-associated toxicities. This work would invariably help practicing oncologists to effectively manage the unique toxicities of FGFR inhibitors.

## 1. Introduction

Fibroblast growth factors (FGFs) facilitate a myriad of physiological functions ranging from early embryogenesis, morphogenesis, and organ formation [1]. The expanding knowledge on the biological activity of FGF uncovered their role in glucose metabolism, bile acid, and phosphorous homeostasis mediated by binding to FGF receptor (FGFR) via klotho co-receptor or heparin cofactor [2,3]. The FGF/FGFR pathway is also known to play an important role in cellular migration, mitogenesis, and cell death, which implicates its role in oncogenic pathways [4]. Understandably, dysregulation of FGF signaling and FGFR aberrations resulted in various congenital disorders, metabolic derangements, and most importantly, cancer. Notably, various *FGFR* gene aberrations have been implicated in human malignancies [5]. For instance, *FGFR* gene amplifications or FGF receptor overexpression have been implicated in the pathogenesis of breast cancer, gynecological malignancies, gastrointestinal malignancies—especially gastric—and hepatocellular carcinoma [5]. Alternatively, constitutive FGFR pathway activation by activating mutations of the *FGFR* gene or reduced dependence on FGF ligands has been implicated as one of the drivers for tumorigenesis. Such *FGFR* gene mutations were implicated in endometrial and urothelial carcinomas [5]. In addition, loss of function mutations in the *FGFR2* gene (*FGFR2^D530N^* and *FGFR2^A648T^*) were implicated in melanoma [6]. Furthermore, two types of fusion aberrations were demonstrated in human malignancies—chromosomal translocations implicated in hematological malignancies including myeloproliferative neoplasms and T-cell lymphoma, whereas fusion rearrangements were associated with solid tumors such as intrahepatic cholangiocarcinoma [7]. Translocations in *FGFR* gene resulting in fusion aberrations have been a matter of interest given their prevalence in various malignancies such as intrahepatic cholangiocarcinoma, muscle invasive urothelial carcinoma, rhabdomyosarcoma, and glioblastoma [8]. Finally, some cancers are known to produce enormous amounts of FGF ligands leading to continuous FGFR pathway activation via autocrine or paracrine manner. This autocrine or paracrine-mediated unopposed FGFR pathway has been well studied in tumor-induced osteomalacia [9]. Table S1 summarizes FGFR aberrations in human malignancies.

Given the implications of the aberrant FGF pathway in oncogenesis, FGFR inhibitors have become an attractive therapeutic target. However, mixed results were reported in early-phase clinical trials that evaluated targeted therapies against the FGF/FGFR pathway highlighting the complex interplay of FGFR signaling in tumorigenesis [10]. Moreover, FGFR inhibitors were shown to cause untoward unique side effects due to the blockade of various FGF signaling physiological pathways. Herein, we sought to summarize the physiology of the FGF/FGFR signaling pathway and its aberrations driving oncogenesis. Moreover, we detailed the possible mechanisms that could lead to FGFR inhibitor resistance, possible side effects, and their management. Many FGFR inhibitors are under clinical investigation for various malignancies; hence, a better understanding of the pathogenesis of toxicities would invariably help us in preventing and managing these toxicities, thereby enhancing their clinical benefit.

## 2. Materials and Methods

Search criteria included the terms: “FGFR”, “FGF pathway”, “FGF oncogenesis”, “FGFR inhibitors”, “FGFR inhibitor resistance”, “FGFR inhibitor side effects”, “side effects”, “phosphorous homeostasis”, “hyperphosphatemia”, “fatigue”, “FGFR inhibitor ocular side effects”, “dysgeusia”, “stomatitis”, “FGFR inhibitor dermatological side effects”, and “diarrhea”. Literature published in English between the years 1985 and October 2020 was included. In addition, we included the data from the abstracts presented at national and international meetings of American Society of Clinical Oncology (ASCO), National Comprehensive Care Network (NCCN), and the European Society of Medical Oncology (ESMO). Management of side effects including hyperphosphatemia, diarrhea, and retinopathy were extrapolated from respective guidelines, which are cited appropriately in respective sections.

## 3. The FGF-FGFR Pathway

The FGF/FGFR family consists of 23 FGF ligands that bind to 5 FGFRs. The FGFR family has five distinct receptors—FGFR1-5, encoded by *FGFR1-4* and *FGFRL1* genes, respectively [11]. FGFs are glycoproteins that are broadly classified into hormonal and canonical subtypes based on their mechanism of action and binding to FGFR. Hormonal FGFs (FGF19, FGF 21, and FGF 23) bind to FGFR in the presence of a klotho protein whereas canonical FGFs bind to FGFR via heparan sulfate glycosaminoglycans and function in an autocrine/paracrine manner. FGFR 1-4 comprise extracellular immunoglobulin domains (Ig I–III), a transmembrane hydrophobic segment, and an intracellular tyrosine kinase domain. Notably, FGFR5 does not have an intracellular tyrosine kinase domain and so lacks receptor tyrosine kinase activity. The five different FGFRs are known to differ in their tissue distribution and affinity to ligands [12]. The interaction of the FGF ligand to FGFR leads to dimerization of the latter, inducing the phosphorylation of FGFR substrate 2 (FRS2). This FRS2 instigates the downstream phosphoinositide-3-kinase (PI3K)/protein kinase B (AKT) and mitogen-activated protein kinase (MAPK) pathways. In addition, activation of the FGFR pathway stimulates protein kinase C via phospholipase C-gamma (PLC-γ) signaling [10]. The FGF–FGFR pathway is involved in various physiological functions depending upon the FGF ligand and the receptor. FGFR1 plays a key role in embryonic development, regulation of cell cycle, wound healing, and phosphorous homeostasis. FGFR2 and FGFR3 are primarily involved in embryonic development, cell cycle regulation, blood vessel formation, and wound healing [13]. While FGFR4 is also involved in embryogenesis, angiogenesis, and tissue repair, it has ligand-specific involvement in glucose homeostasis (FGF19 mediated), bile acid metabolism (FGF19 mediated), and vitamin D homeostasis (FGF23 mediated).

### 3.1. FGFR Pathway in Oncogenesis

**Figure 1:**
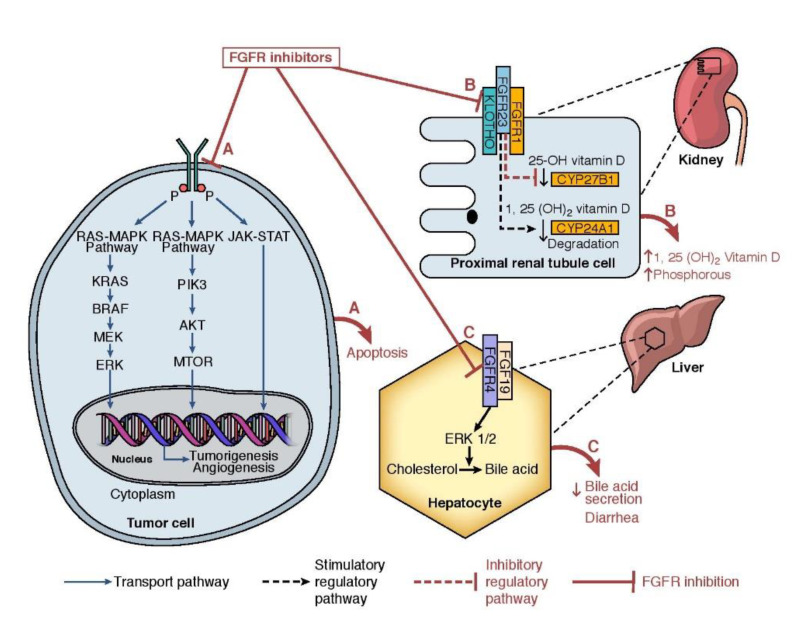
(**A**) Mechanism of action of FGFR inhibitors and FGFR signaling pathway: FGFR inhibitors act by inhibiting the FGFR downstream signaling pathway (**B**) Pathophysiology of FGFR-inhibitor-associated hyperphosphatemia: In kidneys, FGFR inhibitors target FGF23 ligand and FGFR1 receptor complex in the presence of klotho. Activation of the FGF23-FGFR1 pathway leads to inhibition of 25-OH vitamin D activation and degradation of 1, 25 (OH)_2_ vitamin D. FGFR inhibitors block the catabolism of 1, 25 (OH)_2_ vitamin D, and sodium-phosphate co-transporters in the proximal renal tubule cell thereby leading to hyperphosphatemia. (**C**) Pathophysiology of FGFR inhibitor-associated diarrhea: The FGFR4/FGF19/ERK1/2 pathway potentiates the conversion of cholesterol to bile acid in the liver. FGFR inhibitors block the conversion of cholesterol to bile acid thereby leading to altered bile acid metabolism.

Given the ubiquitous nature of FGFRs and their role in the physiological pathways detailed above, numerous pathological conditions including developmental syndromes and malignancies are closely linked to derangement of FGFR signaling. FGF–FGFR pathway aberrancies may result from unopposed autocrine or paracrine activation of FGFRs by FGF ligands secreted by tumor cells or surrounding stromal cells, respectively [14]. In addition, molecular alterations in *FGFR* genes lead to ligand-independent activation of FGFR downstream signaling pathways [14]. A next-generation sequencing (NGS) study that evaluated 4853 tumor specimens showed that FGFR aberrations were identified in 7% of the tumors analyzed [15]. Gene amplifications were the most identified aberrations, present in 66% of the samples, which were often reported in *FGFR1* and *FGFR4*. Fusions were predominantly seen in *FGFR2* and *FGFR3*. These genetic amplification and fusion aberrations activate the intracellular kinase domain, which further activates the downstream MAPK and PI3K/AKT pathways (Figure 1). Interestingly, apart from these genetic aberrations, downstream pathways can also be activated by activated FGFR resulting from the paracrine effect from FGF ligands produced by the tumor cells. This mechanism has been well demonstrated in tumor-induced osteomalacia [9], breast cancer [16,17], and non-small cell lung cancer [18]. FGF ligands are also known to promote tumor growth and proliferation by inducing neo-angiogenesis by indirectly synergizing vascular endothelial growth factor (VEGF) and platelet-derived growth factor pathways (PDGF) [19]. Furthermore, preclinical studies demonstrated the FGFRs crosstalk with other cell surface receptors such as G-protein-coupled receptors or receptor tyrosine kinases. For instance, in vitro studies demonstrated that the crosstalk between FGFR1 and epidermal growth factor receptor (EGFR) can activate AKT and STAT3 signaling in EGFR pathway [20]. A similar crosstalk between FGFR2-IgIIIb and EGFR was noted in breast cancer cell lines [21], opening doors for possible therapeutic interventions with combination therapies.

### 3.2. Targeting the FGFR Pathway

As our knowledge on FGFR aberrations in tumorigenesis of various malignancies continue to evolve, FGFR inhibitors emerged as potential targeted therapy agents. These agents include FGF ligand traps, antibodies that target FGFR, and small-molecule tyrosine kinase inhibitors that block the intracellular tyrosine kinase activity of FGFR. These tyrosine kinase inhibitors can either be selective to FGFR or may be non-selective, having activity against other tyrosine kinase domains such as VEGF and PDGF. While it looks promising to block multiple downstream signaling pathways simultaneously, this could potentially lead to unwanted side effects. Moreover, these tyrosine kinase inhibitors are known to have differential activity on various FGF receptors based on their affinity and minimum inhibitory concentration levels (IC50) (Table 1) [8]. In addition, FGFR inhibitors demonstrated differential effects on various genetic aberrations [8,22]. Table 2 summarizes clinical efficacy of United States Food and Drug Administration approved FGFR inhibitors in urothelial carcinoma and cholangiocarcinoma.

### 3.3. Effects of FGFR Inhibition on Tumor Microenvironment

The tumor microenvironment (TME) has been shown to be an important driver for carcinogenesis. TME predominantly consists of malignant cells and stromal/immune cells. Stromal cells include regulatory T-cells, tumor-associated macrophages, endothelial cells, cancer-associated fibroblasts, and myeloid-derived suppressor cells (MDSCs). Preclinical studies have demonstrated the role of the FGFR signaling in promoting tumor-associated macrophages and MDSCs [27]. FGFR inhibitors, AZD4547, and infigratinib were shown to act on TME leading to disappearance of MDSCs promoting the tumor [27,28]. In addition, FGFR inhibitors demonstrated tumoricidal effects both directly and indirectly by direct tumoricidal effects and by blocking paracrine signaling, respectively [7]. Preclinical studies demonstrated that the FGF2 ligand downregulates the *TP53* gene thereby activating the cancer-associated fibroblasts promoting the tumorigenesis in FGFR aberrations [29]. Infigratinib and ponatinib were shown to upregulate *TP53* activity inducing cell death [29]. Furthermore, FGF2 was shown to promote FGFR1 and FGFR2 signaling leading to proliferation and migration of endothelial cells promoting angiogenesis [30]. This activation of FGF-FGFR signaling was shown to be a contributing factor to VEGF inhibitor resistance prompting the idea of the combination therapies of anti-VEGF agents and FGFR inhibitors [31]. Overall, FGFR inhibitors were shown to express their antitumor activities directly by targeting FGFR alterations and indirectly by altering the TME by regulating the immune environment, angiogenesis, and paracrine effects of FGF ligands.

## 4. Resistance to FGFR Inhibitors

Resistance to FGFR therapy can be attributed to FGFR inhibitor binding site mutations, gatekeeper gene mutations, acquired kinase domains, and the activation of alternate pathways such as epithelial growth factor receptor (EGFR), PI3K, and MEK pathways [32,33,34]. Of these, mutations in gatekeeper genes were shown to be more often associated with FGFR inhibitor resistance. Interestingly, different mechanisms were implicated in developing resistance—for instance, *FGFR1^N546K^* mutation led to increased affinity to adenosine triphosphate (ATP) [35]. This mutation would lead to FGFR inhibitors that act via competitive ATP inhibition. On the contrary, *FGFR1^V561M^* mutation confers FGFR resistance by decreasing the affinity of FGFR inhibitors to the binding site [35]. This leads to augmented levels of auto-phosphorylation, which activates the downstream signaling pathway [36]. Notably, these gatekeeper mutations in FGFR3 (*FGFR3^Y373C;V555M^*) were also shown to confer a 100-fold increase in resistance to various FGFR inhibitors such as AZD4547, AZ8010, and PD173074 [37]. Molecular genetic analysis of circulating tumor cells in biliary tract cancers demonstrated gatekeeper mutations as a potential cause of infigratinib resistance [32]. In addition to gatekeeper mutations, mutations near ATP binding pocket such as *FGFR2^N549H^* resulted in decreased sensitivity to various FGFR inhibitors such as infigratinib, erdafitinib, and pemigatinib [38]. Next-generation sequencing studies from a patient that developed resistance on infigratinib demonstrated p.E565A and p.L617M single-nucleotide variants in the FGFR2 kinase domain [39]. Furthermore, an *in vitro* analysis showed that p.E565A and p.L617M single-nucleotide variants resulted in resistance to other FGFR inhibitors such as erdafitinib, futibatinib, and AZD4547 [39]. It is interesting that these single-nucleotide variants decreased FGFR sensitivity by 2- to 1000-fold. On further Reverse Phase Protein Arrays (RPPA) analysis and Western blot, authors identified that there was an upregulation of phosphorylated ribosomal protein S6, phos-AKT, and phos-mTOR suggesting PI3K/AKT/mTOR signaling pathway activation [39]. Similarly, Fumarola et al. evaluated the role of the irreversible FGFR inhibitor, UPR1376, in blocking FGFR1 phosphorylation in in vitro non-small cell squamous cell carcinoma FGFR1 over-expression cell lines (SQCLC SKMES-1 cells) and FGFR1 amplified cell lines (H1581 cells) [40]. UPR1376 inhibited cell proliferation in both the cell lines including the ones with resistance to infigratinib. The authors found that higher concentrations of UPR1376 were needed in infigratinib-resistant cell lines that had *NRAS* amplification leading to MAPK activation. Interestingly, the addition of the MEK inhibitor, trametinib, potentiated the antitumor effect of UPR1376. This identification of resistance mechanisms and alternate pathway activation opens doors to the concept of combination therapy strategies, especially with FGFR and mTOR inhibitors and FGFR and MAPK pathway inhibitors in FGFR inhibitor resistance. However, it still must be determined if this combination approach would be effective in patients. Interestingly, early clinical data in individual patients showed encouraging results with this combination approach of mTOR inhibitors and FGFR inhibitors in cholangiocarcinoma and hepatocellular carcinoma that had FGFR-inhibitor resistance [39,41]. Recently, Goyal et al. reported that acquired kinase domains of *FGFR2* could lead to resistance to FGFR inhibitors such as infigratinib [34]. While possible resistance mechanisms of FGFR inhibitors still need to be explored, they have been extensively discussed elsewhere [14,42]. Genomic profiling data proposed by Silverman et al. could help us in providing a better insight on possible resistance mechanisms of FGFR inhibitors [43].

Interestingly, preclinical studies have evaluated the combination of FGFR inhibitors and anti-Programmed cell death protein 1 (PD-1) agents showing enhanced tumoricidal effect by enhancing anti-tumor immune activation [44]. Palakurthi et al. [44] demonstrated a survival benefit with the combination of erdafitinib and a PD-1 blockade in a lung cancer mouse model (FGFR2K660N/p53-mutant) as compared to either agent alone. The enhanced antitumor activity with the combination of immune-checkpoint inhibitor and erdafitinib was attributed to decreased expression of PD-1, expansion of T-cell clones, and alternation of tumor microenvironment by immunological changes mediated by erdafitinib. In addition, the non-T-cell inflamed subtype of urothelial carcinoma harboring FGFR3 mutations was found to have low to absent CD8+ T-cells in the tumor microenvironment resulting in resistance to immune checkpoint inhibitor monotherapy [45]. The concept of FGFR3 mutation-driven immunotherapy resistance is exploited by FIERCE-22 phase Ib/II trial that evaluated the combination of vofatamab, a selective inhibitor of FGFR3 in combination with pembrolizumab in metastatic urothelial carcinoma [46]. The trial demonstrated an encouraging prolonged PFS with the combination of pembrolizumab and vofatamab as opposed to pembrolizumab alone. Given these encouraging results, the FGFR inhibitors pemigatinib and derazantinib are currently being evaluated in combination with pembrolizumab (ClinicalTrials.gov Identifier: NCT04003610, FIGHT-205) and atezolizumab (ClinicalTrials.gov Identifier: NCT04045613), respectively, in phase II trials involving patients with advanced urothelial carcinoma.

## 5. Management of FGFR-Inhibitor-Associated Toxicities

Table 3 summarizes the key FGFR-inhibitor-related toxicities observed in clinical trials involving the patients with urothelial carcinoma and cholangiocarcinoma. The most common adverse events include phosphate imbalances, diarrhea, fatigue, and varied dermatological or ocular toxicities, which are discussed in detail in the subsequent sections.

### 5.1. Phosphate Imbalance

Hyperphosphatemia occurred at a rate of 60–76% of patients in the clinical trials involving FGFR inhibitors in various malignancies [22,23,24,25,47,48]. Notably, only a small percentage of these patients experienced grade ≥3 side effect likely due to dose interruptions and aggressive management of hyperphosphatemia. Even though the degree of hyperphosphatemia (phosphate levels >5.5 mg) is as high as 92% in a pooled analysis that included patients with pemigatinib, only 30% of them required phosphate lowering therapy [49]. In a phase II trial that evaluated erdafitinib in urothelial carcinoma, dose reductions and interruptions were seen in 7% and 24% of the patients, respectively [50]. In addition, in the patients with urothelial carcinoma who received infigratinib, hypophosphatemia of any grade and grade 3 was seen in 10% and 7.5% of the patients, respectively [51]. The percentage of phosphate imbalance with FGFR inhibitors seen in the clinical trials underscores the need for effective management of phosphate levels that could translate to minimal dose reductions and interruptions.

#### 5.1.1. Pathophysiology of Hyperphosphatemia

Data from tumor-induced osteomalacia (TIO), familial tumoral calcinosis (FTC), and X-linked hypophosphatemic rickets studies have demonstrated the role of FGFR1 and its ligand FGF23 in tightly regulating phosphate balance in conjunction with the parathyroid hormone [9,13]. FGFR1 receptor is present in proximal renal tubule cell and its ligand FGF23 binds to the receptor in the presence of klotho (Figure 1). This binding of FGF23 in the presence of klotho stimulates the downstream FGFR1 signaling pathway. The activation of the FGFR1 signaling pathway in the proximal renal tubule leads to the inhibition of sodium-phosphate co-transporters (SLC34A1; SLC34A3). The inhibition of phosphate co-transporters limits the phosphate reabsorption in kidneys. In addition, FGF23 blocks the conversion of 25-hydroxyvitamin D to its active form 1, 25-dihydroxyvitamin D thereby limiting the phosphate absorption from intestines. Hyperphosphatemic familial tumoral calcinosis (FTC) is a rare genetic disorder resulting from the mutations in *FGF23, GALNT3,* or *KL* genes [52]. While the *FGF23* gene regulates the FGF23 synthesis in osteoblasts and osteocytes, *GALNT3* and *KL* genes are involved in glycosylation of FGF23 (via ppGalNacT3 protein) and synthesis of alpha-klotho, respectively. Lack of FGF23 or its regulatory proteins (ppGalNacT3 and alpha-klotho) in FTC results in hyperphosphatemia and calcinosis at various sites. FGFR inhibitor toxicity partially mimics FTC resulting in hyperphosphatemia. Calcinosis is not a typical manifestation of FGFR inhibitor-associated toxicity as the action of FGF23 is not completely blocked by FGFR inhibitors. The degree of FGFR1 inhibition depends on the IC_50_ of the FGFR inhibitor (Table 1). Hyperphosphatemia is an on-target effect, and a clinical trial is evaluating the role of escalating doses of pemigatinib until hyperphosphatemia is observed (ClinicalTrials.gov Identifier: NCT02393248). While hyperphosphatemia has directly resulted from the FGFR inhibitors, hypophosphatemia reported in clinical trials of FGFR inhibitors may have resulted from an overcorrection of elevated phosphorous levels from phosphate binders or decreased nutrient intake stemming from other side effects such as stomatitis. Notably, 23% of the patients had grade 3 hypophosphatemia in a phase II trial involving pemigatinib [24]. Hence, effective management strategies are very much needed in handling phosphate imbalances resulting from FGFR inhibitors to prevent unwanted dose interruptions.

#### 5.1.2. Management of Hyperphosphatemia

##### Dietary Modification

Patients on FGFR inhibitors should invariably be educated about dietary modifications, to reduce the risk of developing hyperphosphatemia [53]. In general, phosphorus is ubiquitous, which makes it hard to eliminate it from a regular diet. However, patients are to be provided with necessary handouts that detail the necessary alternative low phosphorous foods. Processed foods and animal foods are known to have high phosphorous content as compared to plant-derived food [54,55]. For instance, dairy products can be replaced with unenriched rice milk. Similarly, frozen fruit pops are to be preferred to ice cream. Interestingly, a cross-over study involving chronic kidney disease stage 3 and 4 demonstrated that patients who consumed a plant-based diet had lower levels of FGF23 and phosphorous levels [55]. Even in plant-based foods, patients should be advised on avoiding whole grains, black-eyed beans, lentils, nuts, and peanut butter as they are a rich source of phosphorous. Instead, they can rely on green peas, refined grains, and/or honey. It is important to note that dietary modification may not always help in maintaining phosphorous levels within the normal range given its ubiquitous nature. However, dietary intervention should be the first step in the patients whose phosphorous levels rise >25% above baseline within the first week after initiation of FGFR-inhibitor therapy. Along with the dietary interventions, phosphorous-lowering agents should be considered in patients with elevated phosphorous levels (≥7 mg/dL).

##### Phosphate Lowering Therapies

Phosphate-lowering therapies are broadly classified into phosphate binders and phosphaturic agents, which help in decreasing phosphate absorption and increased phosphate elimination from the body, respectively. Commercially available phosphate binders include magnesium hydroxide, calcium-based regimens (carbonate and acetate), iron-based phosphate binders (sucroferric oxyhydroxide, ferric citrate), lanthanum carbonate, and sevelamer. The main challenge with these medications is adherence, which mainly stems from the side effect profile and frequent dosing. Interestingly, in the patients with chronic kidney disease on hemodialysis, the adherence rate of phosphate binders was anywhere in the range of 12–98%, with an average of 52% [56]. This means that only about half of the patients who were started on phosphate binders, maintained adherence. Hence, phosphate binders should be administered at the lowest possible dose and dose escalation should be considered based on the response and desired phosphorous levels. While most of the data regarding phosphate binders come from nephrology standpoint, things get a little complicated in patients on FGFR inhibitors as these agents have propensity to cause diarrhea. In patients who also have FGFR-inhibitor-associated diarrhea, lanthanum and calcium-based regimens are preferred over magnesium-based regimens, sevelamer, and sucroferric oxyhydroxide as the latter can exacerbate the diarrhea caused by FGFR inhibitors. Similarly, lanthanum is preferred over calcium-based regimens in patients with baseline hypercalcemia and who are at high risk of vascular calcifications. Notably, each of these agents has unique administration preferences. For instance, lanthanum should be taken immediately after meals while sevelamer needs to be administered in the middle of the meals. Iron-based regimens (sucroferric oxyhydroxide) have very limited systemic absorption (limiting systemic toxicity) and are active in all gastric pH levels [57]. Patients on iron-based regimens should be educated about the possibility of black-tarry stools. To limit the toxicity of phosphate binders, it is advisable that they should be initiated at the lowest possible dose. Table 4 summarizes the initial preferred dose and the maximum tolerated dose of phosphate lowering agents [58]. After the dose escalation of the phosphate binder, one can consider adding acetazolamide. It is important to note that acetazolamide is contraindicated in patients with advanced liver dysfunction and so it should be avoided in patients with extensive liver metastases and in extensive intrahepatic cholangiocarcinoma with compromised liver function.

##### When to Consider a Dose Reduction or Dose Interruption?

Phosphate level between 7 to 9.9 mg/dL (3.4–4.5 mg/dL) is considered grade 3 adverse event in clinical trials that evaluated FGFR inhibitors. Though there is no optimal cut off phosphate values to consider a dose reduction of the FGFR inhibitor, dose reduction should be considered once the phosphate levels reach ≥7 mg/dL (on two separate occasions) or one episode of ≥10 mg/dL despite optimization of diet and phosphate binding agents. Phosphate level is to be repeated in a week after the dose reduction and if phosphate is <5.5 mg/dL, dose escalation to regular dose can be considered. If phosphate level remains elevated to >7 mg/dL, a second dose reduction or FGFR inhibitor can be considered at this point. Drug re-initiation at a reduced dose can be considered when phosphate is <5.5 mg/dL. Permanent discontinuation of the FGFR inhibitor is to be considered if phosphate level is >10 mg/dL after one dose interruption (Table 5).

### 5.2. Fatigue

Fatigue is a commonly reported (32–71%) adverse event in FGFR-inhibitor clinical trials [22,23,51,59,60]. Despite being one of the common side effects, its pathophysiology is not quite well understood. Possible explanations for fatigue include drug-related mineral and electrolyte imbalances or other unknown effects of the drug, disease-related factors (due to underlying cancer by itself), or patient-related factors such as psychological issues that may be linked to cancer diagnosis. Fatigue in patients receiving FGFR inhibitors should be evaluated by obtaining a detailed metabolic, mineral, and complete blood count. In patients with newly diagnosed anemia, a detailed workup must be performed by obtaining reticulocyte count, ferritin, thyroid panel, and total-iron binding capacity. In addition to correcting underlying biochemical abnormalities, patients should be encouraged to optimize nutrition and sleep and participate in mental relaxation and exercise programs [61]. In patients with grade ≥3 drug-induced fatigue, it is reasonable to consider dose reduction.

### 5.3. Diarrhea

Diarrhea is one of the commonly reported side effects in FGFR-inhibitor clinical trials with the incidence ranging anywhere from 15% to 60%, depending upon the degree of FGFR4 inhibition [22,23,26,60]. Pemigatinib has a weaker effect on FGFR4 with IC50 of 30 nM while erdafitinib has stronger activity on FGFR4 with IC50 of 5.7 nM. The incidence of diarrhea in phase II trials of pemigatinib and erdafitinib was 37% and 51%, respectively [49,50]. Notably, infigratinib resulted in a lower incidence of diarrhea of any grade (15%) in a phase II trial [22]. The lower incidence of diarrhea with infigratinib may be attributed to its limited activity of the FGFR4 receptor. Mouse models demonstrated that bile acids stimulate the FGF19/FGFR4/ERK1/2 signaling pathway, which in turn does cause feedback inhibition of bile acid synthesis in a paracrine and autocrine manner [62,63]. Hence, FGFR-inhibitor-induced blockade of the FGF19/FGFR4 pathway leads to altered bile acid metabolism (Figure 1). Altered bile acid balance is known to cause increased mucosal permeability, peristalsis, and water secretion to intestine via adenylate cyclase and GPBAR1 activation [64].

#### Management of FGFR-Inhibitor-Induced Diarrhea

Patients are to be encouraged to optimize fluid intake, and probiotics can be considered for those who tolerate them. Based on the underlying clinical condition, patients should be advised on liberalizing salt and sugar intake (but limiting a phosphorous-rich diet as detailed above) [65]. Other causes of diarrhea including dietary or infectious etiology should be considered. In grade 1 diarrhea, loperamide 4 mg is to be initiated at the onset of symptoms and can be administered (at the dose of 2 mg) with each stool until the patient achieves a diarrhea-free period of 12 h. Patients should be advised that the loperamide dose should not exceed 20 mg/day. If diarrhea progresses to grade 2, a detailed evaluation for electrolyte abnormalities and appropriate correction is warranted. If the grade 2 diarrhea continues beyond day 2, despite supportive measures and loperamide, the FGFR inhibitor withholding should be considered. The FGFR inhibitor can be restarted once diarrhea resolves or decreases to grade 1 with minimal supportive care. Though not seen often, in grade 3 diarrhea, the FGFR inhibitor should be withheld, and patients are to be admitted to a medical facility for aggressive fluid and electrolyte resuscitation. Other underlying causes for diarrhea are to be ruled out especially if grade 3 diarrhea occurs in FGFR inhibitors with low FGFR4 activity. The FGFR inhibitor may be restarted once diarrhea is resolved with minimal supportive care. A dose reduction is a reasonable option especially in patients whose grade 3 diarrhea persists more than 3 days. The FGFR inhibitor should be permanently discontinued in the event of grade 4 diarrhea and patients should be hospitalized for aggressive supportive care.

### 5.4. Dermatologic Toxicities

**Figure 2:**
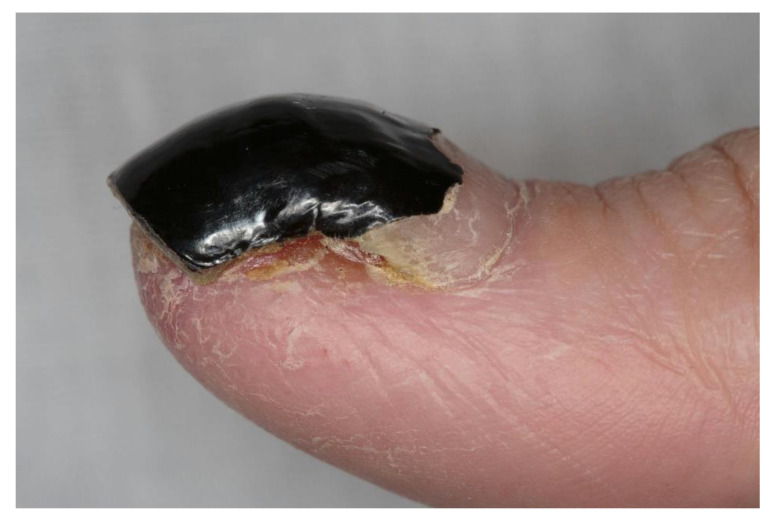
Paronychia in a patient on futibatinib (patient consent obtained).

FGFR-inhibitor-associated dermatologic toxicities such as hand–foot syndrome, hair loss, nail bed infections, onycholysis, dry skin, xerostomia that often leads to altered taste, and stomatitis have been reported in clinical trials and case series [23,60,66,67,68,69]. Among these toxicities, stomatitis is reported commonly at an incidence ranging from 20% to 40% [68,70] and is the first dermatological toxicity seen with FGFR inhibitors. In a phase II trial of pemigatinib, dose reduction and interruptions related to dermatologic toxicity were seen in 3% and 7% of the patients, respectively [24]. A higher percentage of patients (16%) had undergone dose reductions in a phase II trial of erdafitinib in patients with metastatic urothelial carcinoma [23]. In patients with cholangiocarcinoma, alopecia of typically grade 1 and 2 was seen in 24–46% of the patients with FGFR inhibitors [22,24]. Similarly, 30–40% of the patients with urothelial carcinoma experienced grade 1 or 2 alopecia in phase II trials of FGFR inhibitors [23,51]. Alopecia is typically noticed after a couple of months of FGFR inhibitor therapy. Paronychia was reported in 5–17% of the patients with the use of FGFR inhibitors [23,24] (Figure 2).

Patients are to be instructed to keep skin moist, the use of emollients is encouraged, and they are advised to seek medical evaluation for onycholysis to rule out any underlying fungal infection. In all grade ≥3 skin-related side effects, and if symptoms do not get better with emollients, high-potent steroid creams, and lidocaine creams, early dermatology referral is warranted [71]. For dry mouth and dysgeusia, the use of mucosal lubricants and sialagogues such as pilocarpine cevimeline use should be encouraged. Patients should be advised on seeking immediate medical attention in the event of stomatitis. In the event of stomatitis, patients should be instructed on maintaining oral hygiene, avoiding spicy food, and using ice chips (cryotherapy). Non-alcoholic mouth washes that contain sucralfate, doxycycline, and or steroid can also be tried in addition to non-pharmacologic intervention [71]. These patients with stomatitis should be examined for any source of infection, and anti-microbial prophylaxis or treatment may be initiated as clinically warranted. The FGFR inhibitor should be withheld in the event of grade ≥3 stomatitis and other dermatological side effects and can be restarted at a lower dose (one dose reduction) once symptoms resolve to grade ≤1. In the event of grade ≥3 toxicity on the first dose reduction, therapy should be withheld until grade 1 when a second lowered dose may be tried based on the clinical picture and patient preference. The FGFR inhibitor should be permanently discontinued in the event of grade ≥4 side effect or if the patient has persistent grade ≥3 side effect even after two dose reductions. National Comprehensive Cancer Network (NCCN) task force recommendations and dermatology consensus guidelines provide an excellent resource for the management of chemotherapy-induced alopecia, paronychia, and stomatitis [71,72]. Patients should be encouraged that alopecia is resolved on therapy discontinuation and minoxidil 5% and high-potency topical steroids are reasonable options for prophylactic and therapeutic use. The management of FGFR-inhibitor-associated dermatological toxicities is discussed in detail elsewhere [73].

### 5.5. Ocular Toxicities

FGFR inhibitors were associated with a unique ocular side effect profile—central serous retinopathy and retinal detachment have been reported in clinical trials [59,60,67]. For instance, central serous retinopathy was reported in 21% of patients (≥grade 3 side effects in 3%) in a phase II trial of erdafitinib in metastatic urothelial carcinoma [23]. Other ocular side effects reported in clinical trials were dry eyes (22% with pemigatinib [24], 19% with erdafitinib in phase II trials [23]) and cataract (6% with erdafitinib) [23]. In addition, a retrospective analysis noted corneal epithelial dysmaturation as one of the potential ocular toxicities stemming from FGFR inhibition [70].

Given the seriousness of ocular side effects, it is prudent to have immediate ophthalmology consultation in the event of vision changes such as blurry vision or floaters. Ophthalmic examination is recommended at baseline prior to initiating FGFR inhibitors. The FGFR inhibitor should immediately be discontinued in the event of grade ≥3 toxicity. Notably, serous retinopathy was found to be reversible in patients when the FGFR inhibitor was discontinued [74]; therefore, early identification of serous retinopathy and timely discontinuation of the FGFR inhibitor is the key. Patients with blurry vision would more likely benefit from monthly ophthalmology evaluation for early diagnosis and management of serous retinopathy. In grade ≤3 ocular toxicities that were resolved after 4 weeks following onset, the FGFR inhibitor can be restarted at a lower dose under the close supervision of an ophthalmologist. Dose escalation could be tried if the patient has tolerated reduced-dose FGFR inhibitor for at least two cycles. Consideration should be given to permanently discontinue the FGFR inhibitor in the event of a grade ≥2 ocular side effect at a reduced dose or any grade ≥4 ocular side effect.

## 6. Conclusions

FGFR inhibitors are a unique class of emerging targeted therapies that have shown promising results in tumors harboring *FGFR* aberrations. Currently, at least 89 studies (six phase III studies) are actively recruiting patients to analyze the effects of FGFR inhibitors in various malignancies [clinicaltrials.gov]. Given this expanding role of FGFR inhibitors in cancer care, a thorough knowledge on their unique side effects will aid in preventing unnecessary dose reductions and interruptions. It is important that patients be educated about these FGFR-inhibitor-related unique side effects and possible preventive mechanisms. Along with effective management of drug-related toxicities, a special focus is to be made on expanding our knowledge on resistance to FGFR-inhibitors. As determined in preclinical models, combination regimens such as synergistic inhibition of FGFR inhibitors and mTOR or MAPK pathway inhibitors may further be evaluated to bypass the resistance mechanisms.

## Figures and Tables

**Table 1:** Half- maximal Inhibitory Concentration (IC50) of selective FGFR inhibitors on tyrosine kinase FGF receptors.

FGFR inhibitor	FGFR1	FGFR2	FGFR3	FGFR4
Pemigatinib (INCB054828)	0.4 nM	0.5 nM	1 nM	30 nM
Erdafitinib (JNJ-42756493)	1.2 nM	2.5 nM	3 nM	5.7 nM
Infigratinib (BGJ398)	0.9 nM	1.4 nM	1.0 nM	60 nM
Derazantinib (ARQ 087)	4.5 nM	1.8 nM	4.5 nM	34 nM
Futibatinib (TAS 120)	3.9 nM	1.3 nM	1.6 nM	8.3 nM

nM: nanomole.

**Table 2:** Clinical efficacy of selected small-molecule tyrosine kinase inhibitors targeting the FGFR pathway.

FGFR Inhibitor	Study	Significance/Outcome						
		Complete Response	Partial Response	Stable Disease	Progressive Disease	DCR	Median PFS	Median OS
Erdafitinib 8 mg/day	Loriot Y et al. [23] (*n* = 99) Phase II, urothelial carcinoma	3%	37%	39%	18%	79%	5.5 m	13.8 months
Pemigatinib 13.5 mg/day, 2 weeks on and 1 week off	Abou-Alfa G et al. [24] (*n* = 107 ^#^; 20 ^; 18 **) Phase II, CC	3% ^#^ 0% ^ 0% **	33% ^#^ 0% ^ 0% **	47% ^#^ 40% ^ 22% **	15% ^#^ 35% ^ 61% **	83% ^#^ 0% ^ 0% **	6.9 m 2.1 m 1.7 m	21.1 months 6.7 months 4.0 months
Infigratinib 125 mg/day, 3 weeks on and 1 week off	Javle M et al. [25] (*n* = 71) ^#^, Phase II, CC	0%	25%	58%	12%	83%^#^	6.8 m	12.5 months
Futibatinib 20 mg/day	Furuse J et al. [26] (*n* = 67) ^#^, Phase II, CC	1.5%	36%	46%	17%	82%	7.2 m	-

Abbreviations: US FDA: United States Food and Drug Administration; CC: Cholangiocarcinoma; DCR: Disease control rate; PFS: Progression free survival; OS: Overall survival; ^#^ Represent patients harboring FGFR2 fusions or rearrangements (*n* = 107); ^ Represents patients with other FGF/FGFR genetic alterations; ** Represents patients with no FGF/FGFR genetic alterations.

**Table 3:** Common adverse events observed in selected key clinical trials of FGFR inhibitors.

FGFR Inhibitor	Study	Hyperphosphatemia		Diarrhea		Fatigue		Stomatitis		Ocular Side Effects	
		Grade 1–2	Grade ≥ 3	Grade 1–2	Grade ≥ 3	Grade 1–2	Grade ≥ 3	Grade 1–2	Grade ≥ 3	Grade 1–2	Grade ≥ 3
Erdafitinib	Loriot et al. [23] (*n* = 99) Phase 2	75%	2%	46%	4%	30%	2%	47%	10%	Dry eyes-18% Blurred vision-17%	Dry eyes-1%
	Soria et al. [47] (*n* = 11) Phase 1	64%	-	-	-	45%	-	64%	18%	-	-
Pemigatinib	Abou-Alfa et al. [24] (*n* = 145) Phase 2	55%	0%	34%	3%	31%	1%	27%	5%	Dry eyes-21% Keratitis-1%	Dry eyes-1% Keratitis-1%
Infigratinib	Javle et al. [25] (*n* = 71) Phase 2	56%	16%	12%	3%	33%	3%	24%	6%	Dry eyes-21%	-
Derazantinib	Mazzaferro et al. [48] (*n* = 29) Phase 1/2	66%	10%	21%	-	35%	-	-	-	Eye toxicities *-34%	Eye toxicities *-7%

* Eye toxicities include blepharitis, dry eyes, blurred vision, diplopia, keratitis, and corneal disorder. Dose interruption and reduction due to eye toxicity: 24%.

**Table 4:** Dosing considerations of phosphate lowering agents.

Drug	Initial Dose	Max. Recommended Dose
Calcium carbonate	500 mg (chewable) or 600 mg (elemental) tablets 3 times/day	500 mg (chewable) or 600 mg (elemental) tablets 3 times/day
Lanthanum carbonate	500–750 mg three times/day	1000 mg three times/day with meals (taken immediately after meals)
Sevelamer hydrochloride; Sevelamer carbonate	800 mg three times/day	2400 mg three times/day (also available at 1600 mg three times/day) (to be taken in between meals)
Sucroferric oxyhydroxide	500 mg three times/day with meals	2 g per day
Acetazolamide	250 mg once a day	250 mg twice or three times/day *

* Should be avoided in severe liver dysfunction.

**Table 5:** Guidance for the management of FGFR-inhibitor-associated hyperphosphatemia.

Phosphorous Levels	Grade	Intervention *
Sharp rise in phosphorous levels by 25% at the first check; 3.5–5.5 mg/dL	Grade 1	− Reinforce on low-phosphate diet
5.5–6.9 mg/dL	Grade 2	− No dose adjustments for FGFR inhibitor − Consider starting phosphate binder at a lowest possible dose and re-check phosphorous levels in 1 week − Dose escalation of phosphate binder or addition of phosphaturic agent, acetazolamide should be considered if phosphorous levels remain elevated after 7 days of initial intervention
7–9.9 mg/dL	Grade 3	− Maximum recommended dose of phosphate binder in combination with phosphaturic agent, acetazolamide 250 mg twice or thrice a day. − Recheck levels in 2 weeks; if still ≥7 mg/dL; consider dose interruption of FGFR inhibitor for 2 weeks − Consider restarting FGFR inhibitor at usual dose and if phosphorous levels recurs at ≥7 mg/dL, consider first- or second-dose reduction when phosphorous levels reach <7 mg/dL.
≥10mg/dL	Grade 4	− Hold FGFR inhibitor (dose interruption) − Start the patient on maximum recommended dose of phosphate binder in combination with phosphaturic agent, acetazolamide 250 mg twice or thrice a day. − Reassess phosphorous levels every 2 weeks − Consider first- or second-dose reduction when phosphorous levels reach <7 mg/dL. − Consider permanent discontinuation of FGFR inhibitor if phosphorus level >10 mg/dL after two dose reductions

* Phosphorous levels should be checked on day 4, day 21 of each cycle (for a 4-week cycle); day 4 and day 14 of each cycle (for a 3-week cycle).

## Data Availability

The data presented in this study are available on request from the corresponding author.

## Supplementary Materials

The following are available online at https://www.mdpi.com/article/10.3390/cancers13122968/s1, Table S1: *FGFR* genetic aberrations implicated in various human cancers.

## References

[1] Belov A.A., Mohammadi M. (2013). Molecular mechanisms of fibroblast growth factor signaling in physiology and pathology. Cold Spring Harb. Perspect. Biol..

[2] Razzaque M.S., Lanske B. (2007). The emerging role of the fibroblast growth factor-23-klotho axis in renal regulation of phosphate homeostasis. J. Endocrinol..

[3] Nies V.J.M., Sancar G., Liu W., van Zutphen T., Struik D., Yu R.T., Atkins A.R., Evans R.M., Jonker J.W., Downes M.R. (2016). Fibroblast Growth Factor Signaling in Metabolic Regulation. Front. Endocrinol..

[4] Turner N., Grose R. (2010). Fibroblast growth factor signalling: From development to cancer. Nat. Rev. Cancer.

[5] Dienstmann R., Rodon J., Prat A., Perez-Garcia J., Adamo B., Felip E., Cortes J., Iafrate A.J., Nuciforo P., Tabernero J. (2014). Genomic aberrations in the FGFR pathway: Opportunities for targeted therapies in solid tumors. Ann. Oncol..

[6] Gartside M.G., Chen H., Ibrahimi O.A., Byron S.A., Curtis A.V., Wellens C.L., Bengston A., Yudt L.M., Eliseenkova A.V., Ma J. (2009). Loss-of-function fibroblast growth factor receptor-2 mutations in melanoma. Mol. Cancer Res..

[7] Katoh M. (2016). FGFR inhibitors: Effects on cancer cells, tumor microenvironment and whole-body homeostasis (Review). Int. J. Mol. Med..

[8] Mahipal A., Tella S.H., Kommalapati A., Anaya D., Kim R. (2019). FGFR2 genomic aberrations: Achilles heel in the management of advanced cholangiocarcinoma. Cancer Treat. Rev..

[9] Minisola S., Peacock M., Fukumoto S., Cipriani C., Pepe J., Tella S.H., Collins M.T. (2017). Tumour-induced osteomalacia. Nat. Rev. Dis. Primers.

[10] Dieci M.V., Arnedos M., Andre F., Soria J.C. (2013). Fibroblast Growth Factor Receptor Inhibitors as a Cancer Treatment: From a Biologic Rationale to Medical Perspectives. Cancer Discov..

[11] Itoh N., Ornitz D.M. (2004). Evolution of the Fgf and Fgfr gene families. Trends Genet..

[12] Johnson D.E., Williams L.T. (1993). Structural and functional diversity in the FGF receptor multigene family. Adv. Cancer Res..

[13] Su N., Jin M., Chen L. (2014). Role of FGF/FGFR signaling in skeletal development and homeostasis: Learning from mouse models. Bone Res..

[14] Touat M., Ileana E., Postel-Vinay S., André F., Soria J.C. (2015). Targeting FGFR Signaling in Cancer. Clin. Cancer Res..

[15] Helsten T., Elkin S., Arthur E., Tomson B.N., Carter J., Kurzrock R. (2016). The FGFR Landscape in Cancer: Analysis of 4853 Tumors by Next-Generation Sequencing. Clin. Cancer Res..

[16] Fillmore C.M., Gupta P.B., Rudnick J.A., Caballero S., Keller P.J., Lander E.S., Kuperwasser C. (2010). Estrogen expands breast cancer stem-like cells through paracrine FGF/Tbx3 signaling. Proc. Natl. Acad. Sci. USA.

[17] Zhang L., Kharbanda S., Hanfelt J., Kern F.G. (1998). Both autocrine and paracrine effects of transfected acidic fibroblast growth factor are involved in the estrogen-independent and antiestrogen-resistant growth of MCF-7 breast cancer cells. Cancer Res..

[18] Korc M., Friesel R.E. (2009). The role of fibroblast growth factors in tumor growth. Curr. Cancer Drug Targets.

[19] Lieu C., Heymach J., Overman M., Tran H., Kopetz S. (2011). Beyond VEGF: Inhibition of the fibroblast growth factor pathway and antiangiogenesis. Clin. Cancer Res..

[20] Ferguson H.R., Smith M.P., Francavilla C. (2021). Fibroblast Growth Factor Receptors (FGFRs) and Noncanonical Partners in Cancer Signaling. Cells.

[21] Smith M.P., Ferguson H.R., Ferguson J., Zindy E., Kowalczyk K.M., Kedward T., Bates C., Parsons J., Watson J., Chandler S. (2021). Reciprocal priming between receptor tyrosine kinases at recycling endosomes orchestrates cellular signalling outputs. EMBO J..

[22] Javle M., Lowery M., Shroff R.T., Weiss K.H., Springfeld C., Borad M.J., Ramanathan R.K., Goyal L., Sadeghi S., Macarulla T. (2018). Phase II Study of BGJ398 in Patients With FGFR-Altered Advanced Cholangiocarcinoma. J. Clin. Oncol. J. Am. Soc. Clin. Oncol..

[23] Loriot Y., Necchi A., Park S.H., Garcia-Donas J., Huddart R., Burgess E., Fleming M., Rezazadeh A., Mellado B., Varlamov S. (2019). Erdafitinib in Locally Advanced or Metastatic Urothelial Carcinoma. N. Engl. J. Med..

[24] Abou-Alfa G.K., Sahai V., Hollebecque A., Vaccaro G., Melisi D., Al-Rajabi R., Paulson A.S., Borad M.J., Gallinson D., Murphy A.G. (2020). Pemigatinib for previously treated, locally advanced or metastatic cholangiocarcinoma: A multicentre, open-label, phase 2 study. Lancet Oncol..

[25] Javle M., Kelley R.K., Roychowdhury S., Weiss K.H., Abou-Alfa G.K., Macarulla T., Sadeghi S., Waldschmidt D., Zhu A.X., Goyal L. (2018). LBA28—Updated results from a phase II study of infigratinib (BGJ398), a selective pan-FGFR kinase inhibitor, in patients with previously treated advanced cholangiocarcinoma containing FGFR2 fusions. Ann. Oncol..

[26] Furuse J., Goyal L., Meric-Bernstam F., Hollebecque A., Valle J.W., Morizane C., Karasic T.B., Abrams T.A., Kelley R.K., Cassier P.A. (2020). 116MO Efficacy, safety, and quality of life (QoL) with futibatinib in patients (pts) with intrahepatic cholangiocarcinoma (iCCA) harboring FGFR2 fusions/rearrangements: FOENIX-CCA2. Ann. Oncol..

[27] Holdman X.B., Welte T., Rajapakshe K., Pond A., Coarfa C., Mo Q., Huang S., Hilsenbeck S.G., Edwards D.P., Zhang X. (2015). Upregulation of EGFR signaling is correlated with tumor stroma remodeling and tumor recurrence in FGFR1-driven breast cancer. Breast Cancer Res..

[28] Liu L., Ye T.H., Han Y.P., Song H., Zhang Y.K., Xia Y., Wang N.Y., Xiong Y., Song X.J., Zhu Y.X. (2014). Reductions in myeloid-derived suppressor cells and lung metastases using AZD4547 treatment of a metastatic murine breast tumor model. Cell Physiol. Biochem..

[29] Procopio M.G., Laszlo C., Al Labban D., Kim D.E., Bordignon P., Jo S.H., Goruppi S., Menietti E., Ostano P., Ala U. (2015). Combined CSL and p53 downregulation promotes cancer-associated fibroblast activation. Nat. Cell Biol..

[30] Oladipupo S.S., Smith C., Santeford A., Park C., Sene A., Wiley L.A., Osei-Owusu P., Hsu J., Zapata N., Liu F. (2014). Endothelial cell FGF signaling is required for injury response but not for vascular homeostasis. Proc. Natl. Acad. Sci. USA.

[31] Bertolini F., Marighetti P., Martin-Padura I., Mancuso P., Hu-Lowe D.D., Shaked Y., D’Onofrio A. (2011). Anti-VEGF and beyond: Shaping a new generation of anti-angiogenic therapies for cancer. Drug Discov. Today.

[32] Goyal L., Saha S.K., Liu L.Y., Siravegna G., Leshchiner I., Ahronian L.G., Lennerz J.K., Vu P., Deshpande V., Kambadakone A. (2017). Polyclonal Secondary FGFR2 Mutations Drive Acquired Resistance to FGFR Inhibition in Patients with FGFR2 Fusion-Positive Cholangiocarcinoma. Cancer Discov..

[33] Zhou Y., Wu C., Lu G., Hu Z., Chen Q., Du X. (2020). FGF/FGFR signaling pathway involved resistance in various cancer types. J. Cancer.

[34] Goyal L., Shi L., Liu L.Y., Fece de la Cruz F., Lennerz J.K., Raghavan S., Leschiner I., Elagina L., Siravegna G., Ng R.W.S. (2019). TAS-120 Overcomes Resistance to ATP-Competitive FGFR Inhibitors in Patients with FGFR2 Fusion-Positive Intrahepatic Cholangiocarcinoma. Cancer Discov..

[35] Yoza K., Himeno R., Amano S., Kobashigawa Y., Amemiya S., Fukuda N., Kumeta H., Morioka H., Inagaki F. (2016). Biophysical characterization of drug-resistant mutants of fibroblast growth factor receptor 1. Genes Cells.

[36] Zhou W., Hur W., McDermott U., Dutt A., Xian W., Ficarro S.B., Zhang J., Sharma S.V., Brugge J., Meyerson M. (2010). A structure-guided approach to creating covalent FGFR inhibitors. Chem. Biol..

[37] Chell V., Balmanno K., Little A.S., Wilson M., Andrews S., Blockley L., Hampson M., Gavine P.R., Cook S.J. (2013). Tumour cell responses to new fibroblast growth factor receptor tyrosine kinase inhibitors and identification of a gatekeeper mutation in FGFR3 as a mechanism of acquired resistance. Oncogene.

[38] Krook M.A., Bonneville R., Chen H.-Z., Reeser J.W., Wing M.R., Martin D.M., Smith A.M., Dao T., Samorodnitsky E., Paruchuri A. (2019). Tumor heterogeneity and acquired drug resistance in FGFR2-fusion-positive cholangiocarcinoma through rapid research autopsy. Cold Spring Harb. Mol. Case Stud..

[39] Krook M.A., Lenyo A., Wilberding M., Barker H., Dantuono M., Bailey K.M., Chen H.-Z., Reeser J.W., Wing M.R., Miya J. (2020). Efficacy of FGFR Inhibitors and Combination Therapies for Acquired Resistance in FGFR2-Fusion Cholangiocarcinoma. Mol. Cancer Ther..

[40] Fumarola C., Bozza N., Castelli R., Ferlenghi F., Marseglia G., Lodola A., Bonelli M., La Monica S., Cretella D., Alfieri R. (2019). Expanding the Arsenal of FGFR Inhibitors: A Novel Chloroacetamide Derivative as a New Irreversible Agent With Anti-proliferative Activity Against FGFR1-Amplified Lung Cancer Cell Lines. Front. Oncol..

[41] Scheller T., Hellerbrand C., Moser C., Schmidt K., Kroemer A., Brunner S.M., Schlitt H.J., Geissler E.K., Lang S.A. (2015). mTOR inhibition improves fibroblast growth factor receptor targeting in hepatocellular carcinoma. Br. J. Cancer.

[42] Facchinetti F., Hollebecque A., Bahleda R., Loriot Y., Olaussen K.A., Massard C., Friboulet L. (2020). Facts and New Hopes on Selective FGFR Inhibitors in Solid Tumors. Clin. Cancer Res..

[43] Silverman I.M., Hollebecque A., Friboulet L., Owens S., Newton R.C., Zhen H., Féliz L., Zecchetto C., Melisi D., Burn T.C. (2020). Clinicogenomic Analysis of FGFR2-Rearranged Cholangiocarcinoma Identifies Correlates of Response and Mechanisms of Resistance to Pemigatinib. Cancer Discov..

[44] Palakurthi S., Kuraguchi M., Zacharek S.J., Zudaire E., Huang W., Bonal D.M., Liu J., Dhaneshwar A., DePeaux K., Gowaski M.R. (2019). The Combined Effect of FGFR Inhibition and PD-1 Blockade Promotes Tumor-Intrinsic Induction of Antitumor Immunity. Cancer Immunol. Res..

[45] Sweis R.F., Spranger S., Bao R., Paner G.P., Stadler W.M., Steinberg G., Gajewski T.F. (2016). Molecular Drivers of the Non-T-cell-Inflamed Tumor Microenvironment in Urothelial Bladder Cancer. Cancer Immunol. Res..

[46] Siefker-Radtke A.O., Currie G., Abella E., Vaena D.A., Rezazadeh Kalebasty A., Curigliano G., Tupikowski K., Andric Z.G., Lugowska I., Kelly W.K. (2019). FIERCE-22: Clinical activity of vofatamab (V) a FGFR3 selective inhibitor in combination with pembrolizumab (P) in WT metastatic urothelial carcinoma, preliminary analysis. J. Clin. Oncol..

[47] Soria J.-C., Strickler J.H., Govindan R., Chai S., Chan N., Quiroga-Garcia V., Bahleda R., Hierro C., Zhong B., Gonzalez M. (2017). Safety and activity of the pan-fibroblast growth factor receptor (FGFR) inhibitor erdafitinib in phase 1 study patients (Pts) with molecularly selected advanced cholangiocarcinoma (CCA). J. Clin. Oncol..

[48] Mazzaferro V., El-Rayes B.F., Droz dit Busset M., Cotsoglou C., Harris W.P., Damjanov N., Masi G., Rimassa L., Personeni N., Braiteh F. (2019). Derazantinib (ARQ 087) in advanced or inoperable FGFR2 gene fusion-positive intrahepatic cholangiocarcinoma. Br. J. Cancer.

[49] Hoy S.M. (2020). Pemigatinib: First Approval. Drugs.

[50] Markham A. (2019). Erdafitinib: First Global Approval. Drugs.

[51] Pal S.K., Rosenberg J.E., Hoffman-Censits J.H., Berger R., Quinn D.I., Galsky M.D., Wolf J., Dittrich C., Keam B., Delord J.P. (2018). Efficacy of BGJ398, a Fibroblast Growth Factor Receptor 1-3 Inhibitor, in Patients with Previously Treated Advanced Urothelial Carcinoma with FGFR3 Alterations. Cancer Discov..

[52] Boyce A.M., Lee A.E., Roszko K.L., Gafni R.I. (2020). Hyperphosphatemic Tumoral Calcinosis: Pathogenesis, Clinical Presentation, and Challenges in Management. Front. Endocrinol..

[53] Brauer A., Waheed S., Singh T., Maursetter L. (2019). Improvement in Hyperphosphatemia Using Phosphate Education and Planning Talks. J. Ren. Nutr. J. Counc. Ren. Nutr. Natl. Kidney Found..

[54] Joshi S., Potluri V., Shah S. (2018). Dietary Management of Hyperphosphatemia. Am. J. kidney Dis. J. Natl. Kidney Found..

[55] Moe S.M., Zidehsarai M.P., Chambers M.A., Jackman L.A., Radcliffe J.S., Trevino L.L., Donahue S.E., Asplin J.R. (2011). Vegetarian compared with meat dietary protein source and phosphorus homeostasis in chronic kidney disease. Clin. J. Am. Soc. Nephrol..

[56] Ghimire S., Castelino R.L., Lioufas N.M., Peterson G.M., Zaidi S.T.R. (2015). Nonadherence to Medication Therapy in Haemodialysis Patients: A Systematic Review. PLoS ONE.

[57] Floege J., Covic A.C., Ketteler M., Rastogi A., Chong E.M., Gaillard S., Lisk L.J., Sprague S.M. (2014). A phase III study of the efficacy and safety of a novel iron-based phosphate binder in dialysis patients. Kidney Int..

[58] Rastogi A., Bhatt N., Rossetti S., Beto J. (2021). Management of Hyperphosphatemia in End-Stage Renal Disease: A New Paradigm. J. Ren. Nutr..

[59] Arkenau H.-T., Saggese M., Hollebecque A., Mathewson A., Lemech C.R., Landers D., Frewer P., Kilgour E., Brooks N. (2014). A phase 1 expansion cohort of the fibroblast growth factor receptor (FGFR) inhibitor AZD4547 in patients (pts) with advanced gastric (GC) and gastroesophageal (GOJ) cancer. J. Clin. Oncol..

[60] Smyth E.C., Turner N.C., Peckitt C., Pearson A., Brown G., Chua S., Gillbanks A., Johnston S.R.D., Tarazona N., Cutts R. (2015). Phase II multicenter proof of concept study of AZD4547 in FGFR amplified tumours. J. Clin. Oncol..

[61] Bower J.E., Bak K., Berger A., Breitbart W., Escalante C.P., Ganz P.A., Schnipper H.H., Lacchetti C., Ligibel J.A., Lyman G.H. (2014). Screening, assessment, and management of fatigue in adult survivors of cancer: An American Society of Clinical oncology clinical practice guideline adaptation. J. Clin. Oncol. J. Am. Soc. Clin. Oncol..

[62] Song K.-H., Li T., Owsley E., Strom S., Chiang J.Y.L. (2009). Bile acids activate fibroblast growth factor 19 signaling in human hepatocytes to inhibit cholesterol 7alpha-hydroxylase gene expression. Hepatology.

[63] Wu A.-L., Coulter S., Liddle C., Wong A., Eastham-Anderson J., French D.M., Peterson A.S., Sonoda J. (2011). FGF19 regulates cell proliferation, glucose and bile acid metabolism via FGFR4-dependent and independent pathways. PLoS ONE.

[64] Mottacki N., Simrén M., Bajor A. (2016). Review article: Bile acid diarrhoea—Pathogenesis, diagnosis and management. Aliment. Pharmacol. Ther..

[65] Salari P., Nikfar S., Abdollahi M. (2012). A meta-analysis and systematic review on the effect of probiotics in acute diarrhea. Inflamm. Allergy Drug Targets.

[66] Arudra K., Patel R., Tetzlaff M.T., Hymes S., Subbiah V., Meric-Bernstam F., Torres-Cabala C., Aung P.P., Nagarajan P., Diab A. (2018). Calcinosis cutis dermatologic toxicity associated with fibroblast growth factor receptor inhibitor for the treatment of Wilms tumor. J. Cutan. Pathol..

[67] Betrian S., Gomez-Roca C., Vigarios E., Delord J.P., Sibaud V. (2017). Severe Onycholysis and Eyelash Trichomegaly Following Use of New Selective Pan-FGFR Inhibitors. JAMA Dermatol..

[68] Nogova L., Sequist L.V., Cassier P.A., Hidalgo M., Delord J.-P., Schuler M.H., Lim W.-T., Camidge D.R., Buettner R., Heukamp L.C. (2014). Targeting FGFR1-amplified lung squamous cell carcinoma with the selective pan-FGFR inhibitor BGJ398. J. Clin. Oncol..

[69] Van Cutsem E., Bang Y.J., Mansoor W., Petty R.D., Chao Y., Cunningham D., Ferry D.R., Smith N.R., Frewer P., Ratnayake J. (2017). A randomized, open-label study of the efficacy and safety of AZD4547 monotherapy versus paclitaxel for the treatment of advanced gastric adenocarcinoma with FGFR2 polysomy or gene amplification. Ann. Oncol..

[70] Bang Y.-J., Van Cutsem E., Mansoor W., Petty R.D., Chao Y., Cunningham D., Ferry D., Landers D., Stockman P., Smith N.R. (2015). A randomized, open-label phase II study of AZD4547 (AZD) versus Paclitaxel (P) in previously treated patients with advanced gastric cancer (AGC) with Fibroblast Growth Factor Receptor 2 (FGFR2) polysomy or gene amplification (amp): SHINE study. J. Clin. Oncol..

[71] Bensinger W., Schubert M., Ang K.K., Brizel D., Brown E., Eilers J.G., Elting L., Mittal B.B., Schattner M.A., Spielberger R. (2008). NCCN Task Force Report: Prevention and Management of Mucositis in Cancer Care. J. Natl. Compr. Cancer Netw..

[72] Rossi A., Caro G., Fortuna M.C., Pigliacelli F., D’Arino A., Carlesimo M. (2020). Prevention and Treatment of Chemotherapy-Induced Alopecia. Dermatol. Pract. Concept..

[73] Lacouture M.E., Sibaud V., Anadkat M.J., Kaffenberger B., Leventhal J., Guindon K., Abou-Alfa G. (2020). Dermatologic Adverse Events Associated with Selective Fibroblast Growth Factor Receptor Inhibitors: Overview, Prevention, and Management Guidelines. Oncologist.

[74] Prensky C., Marlow E., Gupta M., Sales C., Kiss S., D’Amico D.J. (2018). Reversible Macular Lesions in the Setting of Oral Pan-Fibroblast Growth Factor Inhibitor for the Treatment of Bladder Cancer. J. VitreoRetin. Dis..

