# Supplementary material for: FGFR Inhibitors in Oncology: Insight on the Management of Toxicities in Clinical Practice
Source: Cancers (Basel). 2021 Jun 13;13(12):2968. doi: 10.3390/cancers13122968 (PMC8231807; doi:10.3390/cancers13122968)
Supplement: Supplementary file 1 [file cancers-13-02968-s001.zip › cancers-1228926-supplementary.pdf]

cancers

MDPI

Supplementary

FGFR Inhibitors in Oncology: Insight on the Management of Toxicities in Clinical Practice

Anuhya Kommalapati, Sri Harsha Tella, Mitesh Borad, Milind Javle and Amit Mahipal *

Table S1. *FGFR* genetic aberrations implicated in various human cancers.

Gene.	Type of aberration	Cancers	Prognostic importance
FGFR1	Amplification	Breast, small cell lung, non-small cell lung cancer (squamous type), osteosarcoma, head and neck, gastric and gastro-esophageal junction, endometrial and ovarian, Pancreatic ductal, urothelial carcinoma	Invasiveness in breast cancer, early relapse and poor outcome; resistance to endocrine therapy
	Translocations or fusions	Myeloproliferative syndromes, T-cell lymphoma	
FGFR2	Amplification	Gastric, triple-negative breast	Poor prognosis in gastric cancer
	Translocations or fusions	Intrahepatic cholangiocarcinoma, leukemia; muscle-invasive urothelial carcinoma	Promising data on targeting *FGFR2* fusion or rearrangements (pemigatinib is FDA approved)
	Mutations	Endometrial carcinoma	
FGFR3	Amplification	Breast, urothelial carcinoma	
	Translocations or fusions	Urothelial carcinoma, glioblastoma	Promising data on targeting *FGFR3* fusion or mutations (Erdafitinib is FDA approved)
	Mutations	Urothelial carcinoma	
FGFR4	Amplification	Hepatocellular, ovarian	Invasiveness in liver cancer
	fusions	Rhabdomyosarcoma	Sensitive to FGFR-inhibitors
